# Adherence to opioid agonist therapy predicts uptake of direct-acting antivirals in people who use drugs: results from the French national healthcare database (the ANRS FANTASIO study)

**DOI:** 10.1186/s12954-022-00702-9

**Published:** 2022-10-27

**Authors:** Benjamin Rolland, Caroline Lions, Vincent Di Beo, Patrizia Carrieri, Nicolas Authier, Tangui Barré, Jessica Delorme, Philippe Mathurin, François Bailly, Camelia Protopopescu, Fabienne Marcellin

**Affiliations:** 1 Service Universitaire d’Addictologie de Lyon (SUAL), Hospices Civils de Lyon, CH Le Vinatier, Lyon, France; 2 PsyR2 CRNL, UCBL1, INSERM U1028, CNRS UMR5292, Bron, France; 3 Aix Marseille Univ, Inserm, IRD, SESSTIM, Sciences Economiques & Sociales de la Santé & Traitement de l’Information Médicale, ISSPAM, Marseille, France; 4 CHU Clermont-Ferrand, Neuro-Dol, Service de Pharmacologie Médicale, Centres Addictovigilance et Pharmacovigilance, Université Clermont Auvergne, Clermont-Ferrand, France; 5 Service Des Maladies de L’appareil Digestif, CHU Lille, Université de Lille, Lille, France; 6 Service d’hépatologie et d’addictologie, Groupe Hospitalier Nord, Hôpital de La Croix-Rousse, Lyon, France

**Keywords:** Methadone, Buprenorphine, Hepatitis C, DAA, Drug use

## Abstract

**Background:**

Opioid agonist therapy (OAT) is associated with reduced injection, reduced HCV transmission, and more opportunities to initiate hepatitis C virus (HCV) treatment in people who use drugs (PWUD). We aimed to study the extent to which adherence to OAT was predictive of increased uptake of direct-acting antivirals (DAA) in PWUD with chronic HCV infection.

**Methods:**

Using the French national healthcare system database, we targeted PWUD (i.e. with a history of OAT) who had chronic HCV infection and were eligible for DAA during 2014–2016. Adherence to OAT was computed as a time-varying variable expressing the proportion of days covered by OAT receipt, over any six-month interval before DAA receipt. We used a Cox proportional hazards model to estimate the association between adherence to OAT and the rate of DAA uptake after adjustment for age, sex, alcohol use disorder, socioeconomic status, and liver disease severity.

**Results:**

Among the 22,615 persons included in the ANRS FANTASIO study, 3438 (15.2%) initiated DAA during the study period. After multivariable adjustment, adherence to OAT was associated with a higher rate of DAA initiation. However, this association was not linear, and only individuals on OAT for 20% or more of the time in the previous six-month period had a higher rate of DAA initiation (adjusted hazard ratio [95% confidence interval]: 1.28 [1.18–1.38]). Other variables associated with DAA initiation were male sex, older age, cirrhosis or liver cancer, and higher socioeconomic status.

**Conclusions:**

Adherence to OAT is a major predictor of DAA initiation in PWUD living with chronic HCV infection in France. Our results also suggest that even moderate adherence to OAT can facilitate DAA uptake. Adequate HCV training for OAT prescribers together with interventions to ensure adherence to OAT will help improve DAA initiation rates and reach HCV elimination goals.

**Supplementary Information:**

The online version contains supplementary material available at 10.1186/s12954-022-00702-9.

## Introduction

The availability and high effectiveness of direct-acting antivirals (DAA) for chronic hepatitis C virus infection (HCV) [1, 2] have led to new goals for HCV elimination worldwide.

The World Health Organization (WHO) has set a goal of eliminating HCV infection as a public health challenge by 2030, with a targeted 80% reduction in new infections, 65% reduction in mortality, diagnosis of 90% of people infected with HCV, and treatment of 80% of those diagnosed [3]. To achieve this goal, promoting universal screening and universal access to treatment for HCV is essential, as is identifying which factors can foster access to screening and treatment in the most exposed populations, in particular people who use drugs (PWUD). In France, recent estimates showed a decrease in HCV infection prevalence in the general population between 2011 and 2016 from 0.42 to 0.30% [4]. Such a decrease has not been shown yet among PWUD, but modelling predictions suggest that HCV prevalence in the population under the current model of care in France will decrease to 11.6% by 2026 [5].

Since the 1990s, drug policy in France has constantly aimed to provide easier access to opioid agonist therapy (OAT) for PWUD by involving primary care professionals. This has resulted in approximately 80% OAT coverage in this population [6, 7]. Despite this high rate, adherence to OAT varies considerably and may affect their chances of initiating HCV treatment.

Structural and individual barriers to HCV treatment access have been highlighted in PWUD. The primary barrier they face is self-stigma, which is the consequence of layered stigma and discrimination in society and in the health setting [8] which is often underassessed in HCV research.

In this context, one possible way to encourage HCV treatment initiation in PWUD is through innovative models of care where low-threshold OAT would be a less-stigmatizing entry point.

It is important to note that OAT coverage in most countries where OAT is available is lower than in France [9] and OAT programs may tend to enroll the most motivated and adherent PWUD. Moreover, most studies exploring the relationship between OAT receipt and access to DAA [10–14] generally do not explore in real life to what extent adherence to OAT is predictive of access to HCV treatment.

In France, all health expenditures (i.e., drug prescriptions, dispensed drugs, hospital stays, etc.) are collected in the National System of Health Databases (*Système National des Données de Santé*, SNDS), which was developed by the national health insurance system. The SNDS allows the dynamic monitoring of geographic and individual health inequalities and can be used to analyze access to DAA. Using SNDS data, the ANRS FANTASIO study aimed to identify barriers and facilitators of access to DAA in the national population of chronic HCV patients treated with OAT. In a previous study from the project, we showed that in PWUD with chronic HCV infection, women [15], people with untreated alcohol use disorder (AUD) [16], and individuals living in areas with a limited number of hepatology specialists [17], were all more likely to have lower DAA uptake. In the present study, we aimed to investigate to what extent adherence to OAT was associated with a higher rate of DAA uptake in this population.

## Materials and methods

The reporting of the study is based on the Strengthening the Reporting of Observational Studies in Epidemiology (STROBE) statement [18] (see Additional file 1 for the completed STROBE checklist).

### Data source

We used individual data from the SNDS, which collects anonymized data from all health expenditures (primarily reimbursement data for payments for drugs, clinical tests and procedures, as well as data from public and private hospitals) covering 98.8% of the country’s resident population. The SNDS also includes demographic characteristics (including dates of birth and death, and sex), complementary universal health cover status (*Couverture Maladie Universelle complémentaire* , CMU-C) which provides full healthcare expenditure cover for low-income groups and can be considered a proxy of social deprivation), and cover for long-term conditions (*affection de longue durée*, ALD), which provides full cover for individuals registered as having a costly chronic disease from a specific list [19].

In the present study, individuals with chronic HCV infection were identified using the International Classification of Diseases (ICD-10) (B18.2 code for chronic HCV infection). More precisely, persons registered with an HCV-related ALD or HCV-related hospitalization between 2014 and 2016 were considered to have chronic HCV infection.

### Study population

The present study population included all PWUD living in France with at least one OAT receipt—either methadone or buprenorphine—between 2012 and 2016, who were living with chronic HCV infection between 2014 (i.e., the year when DAA became available) and 2016, and who were therefore eligible for DAA treatment during this period.

### Study period

For each participant, the study period covered the baseline (defined as 1 January 2014 for individuals diagnosed with HCV before this date, or the date of HCV diagnosis for the others) to the first DAA reimbursement, or censure on 31 December 2016, or death, whichever came first.

### Study outcome

The study outcome was the time between the baseline and the first DAA-based HCV treatment receipt during the study period. The following DAA were considered: sofosbuvir, simeprevir, daclatasvir, sofosbuvir + ledipasvir, ombitasvir + ritonavir + boosted paritaprevir, and dasabuvir (with or without ribavirin).

### Variables

For each six-month interval of follow-up over the study period, we used a time-varying variable representing adherence to OAT during those six months, computed as the percentage of time on OAT. We cumulated intervals of less than three months at the end of the study period (i.e., before the event or the censoring time) with the previous six-month interval.

The following other variables were included in the analyses as potential correlates of DAA treatment uptake: age (as a continuous variable), sex, AUD status, CMU-C status, and liver disease severity. AUD was defined as meeting at least one of the following criteria: AUD diagnosis during hospitalization for any cause (ICD-10 code F10), ALD for AUD, or any prescription of disulfiram, acamprosate, naltrexone, or nalmefene, which were the only drugs approved for AUD treatment in France during the study period. We used a three-category variable for AUD status as follows: (1) no AUD, (2) untreated AUD, (3) treated AUD. Liver disease severity was characterized by cirrhosis (ICD-10 codes K74.3 to K74.6) or liver cancer (ICD-10 codes C22.0 to C22.4, C22.7 and C22.9). Accordingly, we built a three-category variable: (1) no cirrhosis and no liver cancer, (2) cirrhosis without liver cancer, and (3) liver cancer.

### Statistical analyses

Descriptive statistics (numbers and percentages for categorical variables, mean and standard deviation (SD) for continuous variables) were used to present the characteristics of the study population. We used Cox proportional hazards models to estimate the association between adherence to OAT and time to DAA uptake, after adjustment for the following baseline characteristics: sex, age, CMU-C, liver disease severity and AUD status. The proportional hazards assumption was tested through a graphical assessment of the assumption of non-zero slope in a linear regression of the scaled Schoenfeld residuals as a function of time. Results of the models are provided as unadjusted hazard ratios (HR) and adjusted hazard ratios (aHR) for the univariable and multivariable models, respectively, and their 95% confidence intervals (CI). We performed a second analysis to identify factors associated with adherence to OAT, using a linear mixed-effects multivariable model.

## Results

Between 2014 and 2016, 22,615 PWUD were identified as living with chronic HCV infection. Most were men (n = 17,682; 78.2%), with a mean (SD) age of 46.4 (7.3) years. Among them, 6496 (28.7%) were CMU-C recipients. In terms of AUD status, 18,247 (80.7%) did not have AUD, 3445 (15.2%) had untreated AUD, and 923 (4.1%) had received treatment for AUD. Liver cirrhosis was reported by 776 (3.4%) persons, while 0.8% (n = 189) were diagnosed with liver cancer (Table 1). Most had less than 20% adherence to OAT (n = 17,224; 76.2%) during the first six months of follow-up. Only 356 (1.6%) individuals were adherent to OAT over the entire study period.

**Table 1:** Characteristics of PWUD with chronic hepatitis C and factors associated with DAA uptake, univariable and multivariable Cox regression (ANRS FANTASIO study; 22,615 patients)

Variables	N (%) or	Univariable analyses		Multivariable analysis	
	Mean (SD) at inclusion	HR [95% CI]	*p*-value	aHR [95% CI]	*p*-value
*Sex*					
Male (ref.)	17,682 (78.2)	1		1	
Female	4933 (21.8)	0.79 [0.72–0.86]	< 0.001	0.82 [0.75–0.90]	< 0.001
Age (per 1-year increase)	46.4 (7.3)	1.04 [1.03–1.05]	< 0.001	1.03 [1.03–1.04]	< 0.001
*Receiving CMU-C*					
No (ref.)	16,119 (71.3)	1		1	
Yes	6496 (28.7)	0.82 [0.76–0.88]	< 0.001	0.91 [0.84–0.98]	0.018
*Liver disease severity*					
No cirrhosis and no liver cancer (ref.)	21,650 (95.7)	1		1	
Cirrhosis without liver cancer	776 (3.4)	4.33 [3.83–4.90]	< 0.001	3.99 [3.50–4.54]	< 0.001
Liver cancer	189 (0.8)	3.78 [2.85–5.03]	< 0.001	3.04 [2.27–4.06]	< 0.001
*Alcohol use disorder (AUD)*					
No AUD (ref.)	18,247 (80.7)	1		1	
Untreated AUD	3445 (15.2)	1.06 [0.96–1.17]	0.224	0.92 [0.83–1.01]	0.090
Treated AUD	923 (4.1)	1.08 [0.91–1.27]	0.369	1.06 [0.89–1.26]	0.505
Adherence to OAT*					
Less than 20% (ref.)	17,224 (76.2)	1		1	
20% and more	5391 (23.8)	1.28 [1.19–1.38]	< 0.001	1.28 [1.18–1.38]	< 0.001
Adherence to OAT*					
0% (ref.)	13,081 (57.8)	1			
1–19%	4143 (18.3)	0.94 [0.86–1.03]	0.205	–	–
20–39%	2126 (9.4)	1.35 [1.21–1.51]	< 0.001		
40–59%	1077 (4.8)	1.37 [1.19–1.57]	< 0.001		
60% +	2188 (9.7)	1.12 [0.99–1.26]	0.069		

aHR, Adjusted hazard ratio; CI, confidence interval; CMU-C, complementary universal health coverage status (*Couverture Maladie Universelle – complémentaire*); DAA, direct-acting antivirals; HR, hazard ratio; OAT, opioid agonist therapy; PWUD, people who use drugs; SD, standard deviation

*Time-varying variable, the numbers and percentages are calculated during the first six months of follow-up

The results of the Cox models are presented in Table 1. Among the 22,615 persons included, 3438 (15.2%) initiated DAA treatment during the study period. The relationship between adherence to OAT and rate of DAA uptake was not linear. Individuals with adherence to OAT ≥ 20% in the previous six months had a higher likelihood of DAA uptake (Table 1). In the multivariable Cox model, adherence to OAT ≥ 20% was associated with a higher rate of DAA uptake (aHR [95% CI] = 1.28 [1.18–1.38]). All tested variables, except for AUD status, were significantly associated with DAA uptake. Older patients (1.03 [1.03–1.04] per 1-year age increase) and patients with cirrhosis and liver cancer (3.99 [3.50–4.54] and 3.03 [2.27–4.06], respectively) had a higher likelihood of DAA treatment uptake compared to other patients (Table 1). By contrast, female sex (0.82 [0.75–0.90]) and social deprivation (receiving CMU-C) (0.91 [0.84–0.98]) were associated with lower DAA uptake rates. The results showed no indication that the proportionality of hazards assumption was violated.

Linear mixed-effects multivariable regression showed that factors associated with adherence to OAT were female sex (adjusted regression coefficient [95% CI] = 1.35 [0.57; 2.13]) and younger age (− 0.09 [− 0.13; − 0.04] per 1-year increase). No association was found between receiving CMU-C, liver disease severity, AUD status and adherence to OAT (Table 2).

**Table 2:** Characteristics of PWUD with chronic hepatitis C and factors associated with adherence to OAT in the previous six months, univariable and multivariable mixed effects linear regression models (ANRS FANTASIO study; 22,615 patients)

Variables	Univariable analyses		Multivariable analysis	
	Coef [95% CI]	*p*-value	aCoef [95% CI]	*p*-value
*Sex*				
Male (ref.)	0		0	
Female	1.45 [0.67; 2.23]	< 0.001	1.35 [0.57; 2.13]	0.001
Age (per 1-year increase)	− 0.09 [− 0.13; − 0.05]	< 0.001	− 0.09 [− 0.13; − 0.04]	< 0.001
*Receiving CMU-C*				
No (ref.)	0		0	
Yes	0.75 [0.05; 1.44]	0.035	0.52 [− 0.18; 1.22]	0.147
*Liver disease severity*				
No cirrhosis and no liver cancer (ref.)	0		0	
Cirrhosis without liver cancer	1.23 [− 0.55; 3.00]	0.176	1.79 [− 0.01; 3.59]	0.051
Liver cancer	0.39 [− 2.69; 3.47]	0.804	1.32 [− 1.79; 4.42]	0.406
*Alcohol use disorder (AUD)*				
No (ref.)	0		0	
Yes	− 0.58 [− 1.34; 0.18]	0.132	− 0.77 [− 1.53; 0.01]	0.051

aCoef, Adjusted regression coefficient; CI, confidence interval; CMU-C, complementary universal health coverage status (*Couverture Maladie Universelle – complémentaire*); Coef, regression coefficient; OAT, opioid agonist therapy; PWUD, people who use drugs

## Discussion

This is the first French nationwide study to explore to what extent adherence to OAT in the previous six months influences the rate of DAA uptake. The main result was that the presence of a threshold effect. More specifically, individuals who had received OAT for more than 20% of the time in the previous six months were more likely to initiate DAA. As OAT prescription is monthly in France, 20% of the time corresponds to two or more OAT prescriptions in the previous six months. The second important result is that only 15% of PWUD with chronic HCV infection in our study received DAA treatment between 2014 and 2016, suggesting that treating strategies were suboptimal in this population. Our results are consistent with previous studies conducted in other countries which have already highlighted that OAT access is a major predictor of DAA uptake [11, 20, 21]. Interestingly, women, as demonstrated elsewhere [15], had fewer opportunities to initiate DAA despite tending to be more adherent to OAT. This indirectly suggests that other barriers, unrelated to OAT access and success, may interfere with opportunities for DAA initiation in women. We wonder whether this association between sex and DAA uptake indirectly expresses discrimination experienced by women who use drugs [8]. This discrimination is associated with self-stigma, which is a known predictor of care avoidance. Another important result is that individuals from socially deprived groups (i.e., receiving CMU-C) tended to have lower DAA uptake rates despite having similar adherence to OAT as others. This would suggest—as was the case for women—that other barriers to DAA initiation are involved, such as prioritizing economic concerns over HCV cure. The effect of age and sex on DAA uptake have already been outlined in previous studies [22]. Social deprivation (captured by the CMU-C indicator) was associated with a lower rate of DAA uptake [23]. Unsurprisingly, greater liver disease severity was associated with a higher rate of DAA uptake.

The considerable variability in adherence to OAT, which we found using French administrative data, may be due to high accessibility and coverage of this treatment in the country. Community pharmacies dispense OAT to a variety of patient profiles, including individuals less motivated to take it. Moreover, the entry point for OAT treatment may influence retention, as demonstrated in a randomized trial conducted in France which showed lower retention rates in individuals who were randomized into specialized care settings compared to those randomized into primary care [24].

Overall, our results suggest that strategies to improve adherence to OAT in HCV-infected PWUD should be a key element for improving DAA uptake rates in this population.

The present study had limitations. First, the study population did not include people with no access to the French health insurance system. However, the latter comprise only 1.2% of the national population [10]. Second, we did not have information on drug use, and receiving OAT was used as a proxy for being a PWUD; however, this can be considered an acceptable approximation, given that 80% of the PWUD population in France receive OAT. Third, OAT can be initiated (but not necessarily followed up) in specialized centers whose data are not collected in the French health insurance system database. However, 90% of French OAT prescriptions are delivered in community pharmacies [25], suggesting that the health insurance database captures most of the population receiving OAT.

In conclusion, adherence to OAT is a major predictor of DAA initiation in PWUD living with chronic HCV infection in France. Our results also suggest that even moderate adherence to OAT can facilitate DAA uptake. This indirectly suggests that adherence to OAT should not be neglected in future analyses estimating the association between receiving OAT and access to treatment for any co-occurring condition in PWUD. Adequate HCV training for OAT prescribers together with interventions to ensure adherence to OAT will help improve DAA initiation rates and reach HCV elimination goals.

## Supplementary Information

**Additional file 1.** STROBE Statement—Checklist of items that should be included in reports of cohort studies.

## Data Availability

Data belong to the French Claims database and are not allowed to be shared to other research teams. The datasets generated and/or analysed during the current study are not publicly available because data are not public and access to data is currently possible only to French researchers upon request to SNDS for a specific research project.
